# Turning Fluids into Data for Precision Oncology: A Multidisciplinary Tumor Board Approach to Malignant Pleural Effusions

**DOI:** 10.3390/biomedicines14030673

**Published:** 2026-03-16

**Authors:** Domenico Damiani, Ilaria Girolami, Esther Hanspeter, Christine Mian, Christine Schwienbacher, Johanna Köhl, Stefania Kinspergher, Giovanni Zambello, Francesco Zaraca, Giovanni Negri, Patrizia Pernter, Mohsen Farsad, Sara Gusella, Georgia Levidou

**Affiliations:** 1 Department of Pathology, Provincial Hospital of Bolzano (SABES-ASDAA), Teaching Hospital of Paracelsus Medical University, 39100 Bolzano-Bozen, Italy; 2 Department of Pneumology, Provincial Hospital of Bolzano (SABES-ASDAA), Teaching Hospital of Paracelsus Medical University, 39100 Bolzano-Bozen, Italy; 3 Department of Oncology, Provincial Hospital of Bolzano (SABES-ASDAA), Teaching Hospital of Paracelsus Medical University, 39100 Bolzano-Bozen, Italy; 4 Department of Vascular and Thoracic Surgery, Provincial Hospital of Bolzano (SABES-ASDAA), Teaching Hospital of Paracelsus Medical University, 39100 Bolzano-Bozen, Italy; 5 Department of Radiology, Provincial Hospital of Bolzano (SABES-ASDAA), Teaching Hospital of Paracelsus Medical University, 39100 Bolzano-Bozen, Italy; 6 Nuclear Medicine, Provincial Hospital of Bolzano (SABES-ASDAA), Teaching Hospital of Paracelsus Medical University, 39100 Bolzano-Bozen, Italy; 7 Department of Pathology, Nuremberg Clinic, Paracelsus Medical University, 90419 Nuremberg, Germany

**Keywords:** multidisciplinary tumor board, liquid biopsy, NSCLC, effusions, molecular profiling, cytology, DNA stability, liquid-based cytology, lung adenocarcinoma, next-generation sequencing

## Abstract

**Background**: Malignant pleural effusion (MPE) represents a frequent and clinically challenging manifestation of advanced malignancy, particularly in metastatic non-small cell lung cancer (NSCLC). Its management requires integration of diagnostic imaging, symptom-directed therapeutic strategies, and, increasingly, molecular profiling technologies. Recent advancements in this field based on liquid medium (so-called liquid biopsy) have achieved a significant increase in sensitivity, enhancing our ability to investigate biofluids and suggesting their potential integration into standard diagnostic practices, far beyond the canonical plasma biopsies. Fluid obtained from MPE after cytological sample centrifugation is rich in cell-free DNA and less susceptible to nucleic acid degradation during processing, improving overall diagnostic accuracy. **Methods**: This narrative review summarizes current evidence on the clinical management of malignant pleural effusions in patients with metastatic NSCLC, integrating imaging, procedural management, and molecular profiling from a multidisciplinary tumor board perspective. The primary objective was to synthesize contemporary knowledge with particular attention to the feasibility, reliability, and reproducibility of pleural fluid-based molecular testing. **Results**: MPE poses diagnostic and therapeutic challenges for all members of the multidisciplinary tumor board, traditionally associated with an adverse prognosis. However, recent advances in cytopathology, histopathology, and liquid-based techniques demonstrate that MPE could be an important source of prognostic or predictive information. At the same time, optimal patient management requires careful integration of imaging findings and procedural strategies (such as pleurodesis or indwelling pleural catheters) with individualized systemic therapy selection. Cell-free DNA in pleural effusions is a promising field of exploration and study, potentially suitable for future guideline implementation, after validation in adequately powered studies, contributing to improving patient management, particularly useful in fragile subsets. **Conclusions:** The management of MPE in advanced NSCLC is evolving toward a multidisciplinary, precision-oriented model that integrates clinical evaluation, imaging, procedural interventions, and molecular testing. Liquid biopsy technology has gained enough analytical robustness and clinical feasibility to be a useful tool in routine analysis. Biofluid-based molecular testing may have outstanding potential, contributing to improving patient management, avoiding repetitive procedures, and optimizing the overall efficiency and cost-effectiveness of diagnostic practices. Moreover, collaborative projects among different specialties help in consolidating trust in the tumor board decision-making process.

## 1. Introduction

During the last few years, clinical oncology has witnessed a revolution in therapeutic possibilities. Since the beginning of chemotherapy, toxic compounds have been used to reduce disease burden, with the aim of reducing neoplastic mass, often reaching modest therapeutic benefit and with strong adverse effects. The inability to selectively deliver the therapeutic toxic effect to neoplastic cells represents a major problem of standard chemotherapy. With the advent of precision oncology, a new generation of drugs has been introduced, which can inhibit or selectively kill neoplastic cells that harbor a specific, so-called “targetable” molecular alteration.

The efficacy of this new class of compounds was so impactful that it led to radical debate in the medical community on whether there is a need to establish a new pragmatic classification of neoplastic diseases [1], no longer based on morphological aspects, but on molecular characteristics, on the assumption that those altered genetic profiles are not only responsible for uncontrolled neoplastic cell growth but potentially treated with a selective weapon, able to influence disease outcomes. Thus, molecular biology has gained a pivotal role as a predictive tool of response to therapy, while recent technologies have emerged as new standards in pathology and cytology diagnostics [2].

New molecular analysis technologies allow the use of a liquid source of nucleic acids, laying the foundation of the next revolution in precision oncology, collectively grouped under the term “liquid biopsy” [3,4]. Since potentially any biological fluid (e.g., sputum, cerebrospinal fluid, plasma, and urine) can be used to extract and analyze nucleic acids, an increasing number of reports describing high concordance between tissue and biofluids have been published among the scientific community [5,6,7,8].

Malignant pleural effusion (MPE) at onset represents a defined and frequent presentation of metastatic non-small cell lung cancer (NSCLC), with it being associated with adverse prognosis. Beyond its molecular relevance, MPE represents a complex clinical condition requiring careful diagnostic imaging assessment, symptom-directed interventions, and procedural management (such as thoracentesis, pleurodesis, or indwelling pleural catheter placement). It represents not only a good source of cytological material for morphological diagnosis but also an optimal source of nucleic acids for molecular profiling, obtained without exposing patients to procedures at high risk of adverse events [5,9]. Therefore, its evaluation extends beyond pathology laboratories and involves radiologists, pulmonologists, thoracic surgeons, and medical oncologists in coordinated decision-making processes.

**Figure 1:**
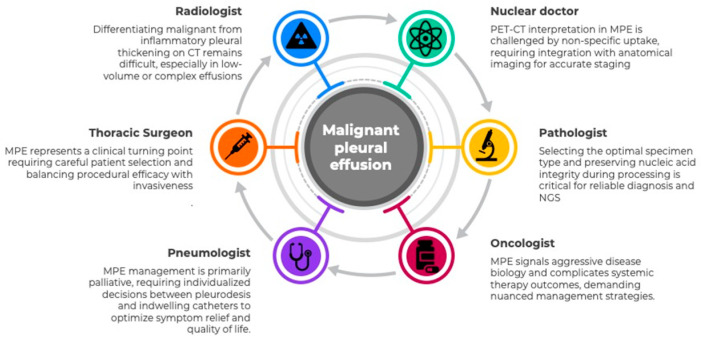
Thoracic tumor board specialties facing MPE.

The tumor board (TB) is the natural setting for deciding multidisciplinary therapeutic approaches in oncological patients and is increasingly influenced by new molecular technologies. However, optimal management of MPE requires integration of radiological staging, procedural strategies for effusion control, systemic treatment planning, and quality-of-life considerations, in addition to molecular profiling. The approach and usefulness of the information gained using these new techniques may be perceived and used in different ways, depending on the TB participant’s discipline. Here we analyze the different approaches, intervention possibilities, and the influence of molecular profiling in MPE for pneumologists, radiologists, surgeons, oncologists, and pathologists, framing MPE as both a therapeutic challenge and a diagnostic opportunity within a multidisciplinary precision oncology model. This multidisciplinary approach is illustrated in Figure 1.

## 2. Methods

The literature search was conducted using PubMed/MEDLINE, with the last search updated in December 2025. The search strategy combined the following keywords and Medical Subject Headings (MeSH) terms: “malignant pleural effusion,” “non-small cell lung cancer,” “NSCLC,” “liquid biopsy,” “cell-free DNA,” “supernatant,” “cytology,” “next-generation sequencing,” “molecular profiling,” and “precision oncology.” Boolean operators (AND/OR) were used to refine the search.

Search criteria were designed to prioritize high-quality evidence, including systematic reviews, clinical practice guidelines, and, when available, large randomized clinical trials. Eligible publications included original research articles (retrospective and prospective cohort studies), randomized clinical trials, systematic reviews, clinical practice guidelines, and consensus statements published in English. Studies focusing exclusively on non-oncologic pleural effusions, case reports with fewer than five patients, and articles lacking molecular or biomarker-related analysis were excluded. Studies published in the grey literature or non-indexed sources were intentionally excluded to ensure methodological consistency.

Reference lists of key publications were also screened to identify additional relevant articles. To minimize the risk of omitting important contributions, findings from the PubMed search were cross-checked and integrated with results obtained through an Elicit query using the prompt: “precision oncology in metastatic NSCLC malignant pleural effusion.” This complementary step allowed the inclusion of pertinent studies that may not have been captured through traditional database searching.

Study quality was not formally assessed using standardized scoring tools, given the narrative design of this review; however, priority was given to peer-reviewed publications with clearly described methodology, defined patient cohorts, and reported analytical performance metrics (e.g., concordance rates, sensitivity, and variant allele frequencies). Particular emphasis was placed on studies reporting sample size, study design, and reproducibility of molecular findings.

Because the purpose of this work is to provide a clinically oriented overview centered on malignant pleural effusions in advanced NSCLC patients—and not to fulfill the methodological requirements of a systematic review—standardized review frameworks (e.g., PRISMA) were not applied. Instead, study selection and synthesis were guided by clinical relevance, strength of evidence, and alignment with the review’s thematic focus. When available, priority was given to studies reporting sample size, concordance rates, and analytical performance metrics relevant to molecular profiling in malignant pleural effusions.

## 3. Measuring the Magnitude: The Pneumologists’ Perspective

Current evidence underscores that MPE management remains primarily either palliative or in a non-curative setting, aiming to relieve dyspnea and optimize quality of life.

Across randomized trials and retrospective analyses, talc pleurodesis—whether administered as slurry via chest tube or poudrage during thoracoscopy—consistently demonstrates the highest success rates for definitive pleurodesis. The Cochrane network meta-analysis ranked talc slurry among the most effective agents, with pleurodesis failure rates estimated at 10–18%, while talc poudrage shows comparable efficacy (OR 0.50 vs. slurry; moderate certainty). In contrast, indwelling pleural catheters (IPCs) without daily drainage exhibit markedly higher failure rates (≈62% at 12 months) but remain valuable for symptom control and outpatient management [10]. Combined approaches—IPC plus talc instillation—improve pleurodesis success compared to IPC alone, as demonstrated in IPC-Plus and TACTIC [11] trials. Dyspnea relief, the primary patient-reported outcome, appears equivalent across all modalities. Both the TIME2 [12] and AMPLE [13] trials, as well as the U.S. veteran cohort [14], confirmed significant improvement in breathlessness following IPC or talc pleurodesis, with no clinically relevant differences between groups. This finding underscores that intervention choice should prioritize logistical and patient-centered factors rather than anticipated symptom benefit.

Adverse event profiles differ substantially. IPCs are associated with higher rates of pulmonary infections (up to 31.3%) and lung entrapment (14.6%) compared to talc pleurodesis (15.4% and 4.4%, respectively) in the U.S. veteran study. Cochrane review findings align, noting IPC-related infection risk, though generally manageable with antibiotics. Talc pleurodesis carries a minimal risk of severe complications such as ARDS, with large meta-analyses reporting negligible incidence. Procedure-related pain and fever occur across interventions but with no significant differences in pooled analyses. Length of stay (LOS) is a critical differentiator. IPC placement enables outpatient management or short admissions (median 1–3 days), whereas talc pleurodesis typically requires longer hospitalization (4–7 days). This has implications for cost and patient preference, particularly in systems prioritizing home-based care [15].

The recent literature emphasizes individualized decision-making. IPCs are favored for patients seeking hospital avoidance or those with non-expandable lung, while talc pleurodesis remains suitable for individuals with longer prognosis or those already hospitalized. Combined strategies may offer a middle ground, enhancing pleurodesis success without prolonging LOS. Evidence supports talc pleurodesis as the most effective method for durable pleural symphysis, while IPCs provide comparable symptom relief with advantages in outpatient care and reduced repeat interventions. Time of intervention of pleurodesis, whether during pleural biopsy or after definite diagnosis, is still a matter of debate across different national guidelines and requires a tailored approach evaluating patient comorbidities and clinical suspicion, especially in case of infectious etiology [16]. No significant mortality differences exist among strategies. Ultimately, treatment selection should integrate patient goals, expected survival, complication risk, and healthcare resource considerations.

## 4. Complementarity Rather than Competition in Diagnostics: How Radiology and Nuclear Medicine Work Together in Pleural Disease

The literature on MPE management reveals no specialty-specific differences between radiologists and nuclear medicine specialists. This absence reflects the collaborative structure of care, not a methodological gap. Imaging follows a complementary, sequential workflow: chest X-ray, US, and MRI contribute to the diagnosis and provide detail for initial assessment and/or local invasion, and PET-CT (nuclear medicine expertise) adds metabolic and staging information, particularly in complex cases.

Treatment decisions are consistently multidisciplinary, involving oncologists, pulmonologists, thoracic surgeons, and palliative care specialists, with imaging specialists contributing diagnostic input rather than directing therapy [17]. Despite this structured approach, significant evidence gaps persist regarding the comparative effectiveness of imaging modalities. Most recommendations rely on expert consensus, not randomized trials, leading to global practice variations driven by institutional resources rather than inter-specialty disagreements.

Radiologists are primarily engaged in the early diagnostic phase, performing initial anatomical assessment through US (ultrasound and echography), CT, and MRI. CT imaging provides essential morphological characterization of pleural effusion, including volume assessment, identification of loculation, pleural thickening patterns (focal versus diffuse), and detection of pleural nodules or masses. However, CT alone cannot reliably distinguish malignant from inflammatory pleural thickening, particularly in tuberculosis-endemic regions, and small-volume pleural metastases may escape detection.

In contrast, nuclear medicine specialists contribute predominantly during staging and treatment response evaluation. FDG-PET provides metabolic information reflecting cellular glucose utilization but suffers from non-specificity, with significant overlap between malignant and inflammatory processes. Fjaellegaard et al.’s systematic review demonstrated that visual/qualitative interpretation of integrated PET-CT by experienced readers shows sensitivity of 89.4% and specificity of 83.1% [18].

This sequence reflects a complementary, stepwise involvement rather than competition, with radiologists providing structural detail and nuclear medicine specialists adding functional and metabolic insights [19]. PET-CT, which integrates expertise from both radiology and nuclear medicine, is associated with enhanced diagnostic and staging accuracy, reinforcing the concept of complementarity. The integration of PET and CT overcomes individual modality limitations through synergistic assessment. CT findings guide PET interpretation by precisely localizing metabolic activity and providing morphological context, while PET adds functional information to characterize ambiguous CT abnormalities.

Recent evidence shows that integrated scoring systems combining metabolic and radiological parameters provide superior diagnostic accuracy compared to single-modality approaches. Yang et al. [20] developed and validated in a cohort of 176 patients with suspected MPE a five-parameter score to differentiate malignant from benign pleural effusions, assigning points for unilateral lung nodules or masses with increased ^18^F-FDG uptake (SUVmax ≥ 2.5), extrapulmonary malignancy, pleural thickening of at least 3 mm with increased uptake (TBR > 1.8), multiple nodules or masses with uptake (SUVmax ≥ 2.5), and pleural effusion uptake (TBR > 1.1). Using a cut-off of four points, the model achieved an AUC of 0.949 with 83.3% sensitivity and 92.2% specificity in the derivation cohort (*n* = 199) and was validated in an independent cohort (*n* = 74) with an AUC of 0.942, sensitivity of 89.7%, and specificity of 88.6%, clearly outperforming individual parameter assessment. Lu et al. [21] proposed a broader system based on eight parameters, with scores ranging from 0 to 15 and a cut-off of six points, enabling risk stratification. Low scores (0–2) were associated with a negative predictive value of 99.4%, supporting non-invasive management, whereas high scores (9–15) had a positive predictive value of 98.3%, justifying direct intervention; intermediate scores (3–8) required invasive confirmation. This model also provided etiology-specific classification, correctly identifying 96.5% of non-tuberculous benign effusions, 94.3% of mesotheliomas, 87.5% of metastatic effusions, and 79.7% of tuberculous effusions. Unlike Yang’s system, which focuses on five parameters and uses a cut-off of four points, Lu’s approach emphasizes risk stratification and etiology prediction, and the two models should not be conflated.

Notably, the reviewed literature revealed no direct comparisons of diagnostic accuracy or reports of conflicting opinions between these disciplines. Diagnostic evaluation is primarily based on CT interpreted by radiologists, whereas staging of potentially resectable disease typically incorporates both MRI—to assess local invasion—and PET-CT for the detection of nodal and distant metastases. Consequently, the role of nuclear medicine specialists expands in proportion to disease complexity and the consideration of aggressive multimodal treatment strategies [22,23].

## 5. Evidence and Evolving Expectations from the Thoracic Surgeons’ Perspective

For thoracic surgeons, MPE is not just fluid—it is a clinical turning point that demands nuanced decision-making. While the primary objective remains palliative—aimed at relieving dyspnea and improving quality of life—surgical strategies must balance procedural efficacy, invasiveness, patient performance status, and anticipated survival. As emphasized by Bertolaccini and colleagues, careful patient selection based on lung re-expansion capability, functional reserve, and prognosis is essential to optimize outcomes [24].

Among available surgical options, Video-assisted Thoracoscopic Surgery (VATS) with talc poudrage stands out as the cornerstone procedure. VATS uniquely integrates diagnostic and therapeutic objectives within a single procedure, enabling direct pleural inspection, targeted biopsies for histological and molecular profiling, and effective pleurodesis, with reported success rates ranging from 80% to 90%. Direct visualization allows the surgeon to break septations, making VATS ideal for multiloculated effusions or when tissue sampling is critical for targeted therapy.

In selected cases—such as trapped lung or refractory pleural effusions—more aggressive surgical approaches, including pleurectomy or decortication, may be considered. However, these procedures are associated with higher morbidity and should be reserved for carefully selected patients with preserved functional status and favorable risk–benefit profiles. In current practice, such approaches are largely confined to highly selected cases of malignant mesothelioma (MPM), and even in this setting, their role is being increasingly questioned, considering recent randomized data [25].

Pleuroperitoneal shunts remain limited by infection risk and mechanical failure [26]. In MPM, international and European guidelines identify VATS as the reference approach for obtaining adequate pleural tissue, ensuring high diagnostic accuracy and reliable histological subtyping, which are crucial for prognostic stratification and treatment planning [27,28].

A recent survey by Scarci et al. [29] revealed striking heterogeneity in surgical expectations and techniques. For initial evaluation, CT scans (92%) and chest X-rays (78%) were most used. VATS pleurodesis was the preferred approach for uncomplicated MPE (68%), while VATS pleurectomy–decortication was favored for multiloculated effusions or trapped lung (60%). Technical details varied: 45% of surgeons routinely used large-bore chest tubes, and suction management ranged from no suction (35%) to low-pressure suction (−2 kPa or less, 37%). Despite these differences, consensus emerged on one point: talc as the sclerosing agent of choice, adopted by 91% of respondents. Desai et al. [30] confirmed talc’s superiority in efficacy and cost-effectiveness, though povidone–iodine is gaining attention as an alternative [29]. For patients with limited life expectancy or inferior performance status, expectations shift toward ultrasound-guided thoracentesis or IPC to minimize invasiveness and hospitalization. These approaches align with broader goals of outpatient care and cost-effectiveness. Conversely, fitter patients with expandable lungs and longer prognosis are candidates for VATS pleurodesis, which can achieve up to 93% complete response.

A particularly concerning finding from the Scarci survey is that more than half of thoracic surgeons reported they were not adhering to formal guidelines, with many considering existing recommendations outdated. This highlights the need for updated, evidence-based protocols that better reflect contemporary surgical practice and evolving oncologic paradigms. Tools like the LENT score—which integrates LDH, ECOG performance status, neutrophil-to-lymphocyte ratio, and tumor type—are helping calibrate expectations and improve prognostic accuracy. Advanced imaging and thoracoscopy further refine decision-making by reducing complications and improving visualization. The observed heterogeneity in practice reflects uncertainty rather than contradiction. Where robust evidence exists—such as talc’s superiority—practice converges. Variability in chest tube size or suction pressures, by contrast, stems from limited comparative data. Similarly, survival expectations ranging from 3 to 12 months are all valid, explained by performance status (Karnofsky < 30 predicts ~1 month vs. >70 predicts ~13 months), tumor biology, and molecular factors [30,31,32].

Looking ahead, future surgical strategies may increasingly integrate local pleural interventions with systemic oncologic therapies, including the investigational use of intrapleural immunomodulatory or targeted agents in conjunction with thoracoscopic procedures. Prospective studies are needed to define optimal timing, patient selection, and synergistic treatment combinations. Until such data become available, thoracic surgeons must continue to navigate a complex clinical landscape shaped by evolving evidence, patient heterogeneity, and the ongoing need to balance effective palliation with the principles of precision oncology.

## 6. Understanding the Scope: Insights from Oncologists

Healthcare professionals’ prognostic expectations for MPE patients centered on survival duration, with considerable convergence across studies. The systematic review by Meriggi et al. [31] reported an average survival of 4–9 months from diagnosis, while Desai et al. [30] reported median survival ranging from 3 to 12 months, and Skok et al. [32] similarly cited a 3–12-month range. These survival estimates were understood to vary significantly based on patient characteristics, including the underlying malignancy, performance status, and tumor characteristics.

Management of MPE is primarily palliative, with treatment goals centered on symptom relief rather than disease modification. Across studies, the dominant expectation is rapid improvement in dyspnea, reflecting the profound impact of breathlessness on patient quality of life. Penz et al. [33] emphasized that interventions aim to prevent recurrence or enable intermittent drainage, underscoring the focus on comfort and functional status rather than curative intent. However, patient heterogeneity complicates outcome prediction. Variability in tumor biology, systemic therapy response, and pleural environment necessitates individualized treatment planning. Ultimately, expectations for MPE management converge on optimizing quality of life through tailored interventions that balance procedural efficacy with patient-specific prognostic factors.

MPE remains a formidable challenge in the management of advanced NSCLC, shaping both prognosis and therapeutic decision-making. Oncologists increasingly recognize MPE not merely as a symptom but as a marker of aggressive disease biology and an immunosuppressive microenvironment. Two recent studies—Wei et al. (2024) [34] and Nishimura et al. (2022) [35]—offer critical insights into systemic therapy outcomes and prognostic implications, highlighting the complexity of treating NSCLC patients with MPE in the era of immunochemotherapy. Nishimura et al. demonstrated that NSCLC patients with pleural effusion undergoing combined immune checkpoint inhibitor (ICI) therapy and chemotherapy had significantly shorter PFS and OS compared to those without effusion (median OS: 16.4 vs. 27.7 months; HR 2.12, *p* < 0.001) [35]. This negative prognostic effect was most pronounced in patients with high PD-L1 expression, suggesting that MPE may attenuate the benefits of immunotherapy even in biomarker-selected populations. Mechanistically, elevated VEGF levels and M2 macrophage predominance within pleural fluid contribute to immunosuppression and tumor progression, reinforcing the need for tailored strategies. In this context, Wei et al. [34] addressed a critical gap by evaluating ICI plus chemotherapy versus chemotherapy alone in NSCLC patients with MPE. Their multicenter retrospective study found a significant improvement in PFS with combination therapy (7.4 vs. 5.7 months; HR 0.594, *p* = 0.008), though OS benefit was limited (34.2 vs. 28.3 months; *p* = 0.317). These findings suggest that while immunochemotherapy can delay disease progression, its impact on long-term survival remains uncertain—possibly due to subsequent treatment lines and competing risks. Importantly, pleurodesis success rates were similar across treatment arms, indicating that systemic therapy does not obviate the need for local control measures. Despite theoretical synergy between anti-VEGF agents and ICIs, Nishimura et al. [35] reported no survival advantage for bevacizumab-containing regimens in patients with MPE (*p* > 0.1). Similarly, pleural interventions such as drainage or pleurodesis did not improve PFS or OS, underscoring the systemic nature of the disease and the limited impact of local procedures on overall outcomes. These observations challenge oncologists to reconsider the utility of adjunctive strategies and highlight the need for innovative approaches, including intrapleural immunotherapy or combined modality regimens.

From an oncologist’s perspective, MPE signifies a high-risk subgroup requiring nuanced management. Current evidence supports immunochemotherapy as the preferred first-line option, yet expectations for OS improvement should remain tempered. Biomarker-driven personalization, integration of antiangiogenic therapy, and exploration of intrathoracic immunomodulation represent promising avenues. Prospective trials are urgently needed to validate these strategies and refine prognostic models incorporating pleural status. MPE significantly impairs patient quality of life, with patients demonstrating low baseline QoL scores across multiple domains [36]. Studies consistently show that MPE patients have significantly worse physical, mental, and emotional health status compared to patients with other types of pleural effusions. Multiple interventions show modest but meaningful improvements in quality of life: talc pleurodesis generally improved quality-of-life measures, including physical and respiratory domains, though mental health benefits were limited; similar gains were observed in Karnofsky performance and dyspnea scores [37,38]. IPC also yielded significant short-term symptom relief and global health improvements, while systematic reviews indicate all interventions offer modest, inconsistent QoL benefits without clear superiority [39,40].

All major interventions provide significant improvement in dyspnea, the most debilitating symptom of MPE [38,39,40], even if the current absence of specific clinical trials poses difficulties in drawing conclusions. The choice between interventions should be individualized based on patient prognosis, performance status, and clinical circumstances rather than QoL outcomes alone, as effectiveness appears similar across modalities, given the poor prognosis (median survival 3–12 months). Treatment should prioritize symptom palliation and maintain quality of life rather than cure [41].

**Figure 2:**
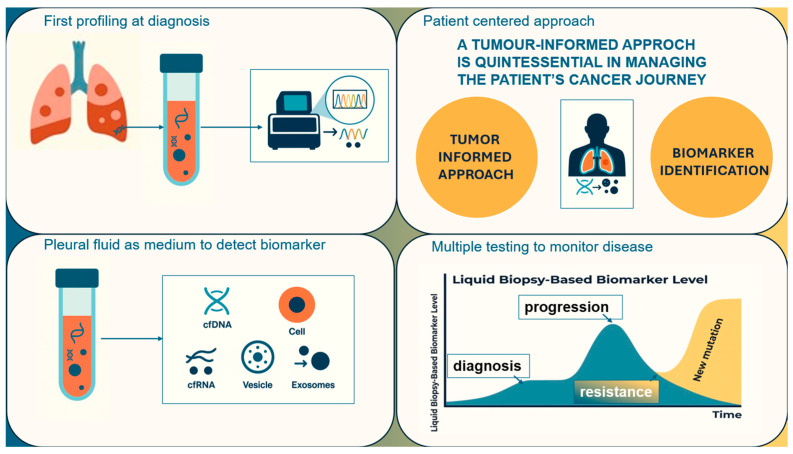
Tumor-informed liquid biopsy workflow in malignant pleural effusion. Overview of a tumor-informed, patient-centered approach using liquid biopsy and pleural fluid analysis to support cancer diagnosis, biomarker detection, and ongoing monitoring. The diagram illustrates initial molecular profiling, identification of tumor derived biomarkers in pleural fluid (cfDNA, cfRNA, shed tumor cells, or circulating tumor cells) as a crucial initial step in malignant pleural effusion. This profiling unlocks a very informative tumor-informed approach which supports repeated liquid biopsy-based testing to monitor disease progression, treatment resistance, and emerging mutations over time.

Upfront molecular profiling at diagnosis, even with the aid of pleural fluid, may enhance therapeutic monitoring, allowing a tumor-informed approach in sequential liquid biopsy application. Dong et al. [42] distinguished two main technological categories: tumor-informed assays that sequence tumor tissue to create personalized minimal residual disease (MRD) panels, and tumor-agnostic assays using fixed panels without prior tumor mutation information. The choice between approaches depended on evidence from clinical trials, investigator preference, cost-effectiveness, patient economics, and tumor tissue availability, although Abbott et al. [43] directly compared tumor-informed liquid biopsy with typical liquid biopsy panels, concluding that tumor-informed approaches provided more robust understanding of therapeutic response and resistance mechanisms than panels alone. Moreover, Krebs et al. [44] noted that although tissue biopsy remained preferred for initial diagnosis, liquid biopsy proved particularly valuable for patients unable to undergo tissue biopsy or when providing more representative views of tumor burden than tissue-based assays. Liquid biopsy complements tissue analysis, especially in advanced lung cancer, as depicted in Figure 2.

Oncologists increasingly view MPE as both a therapeutic challenge and a prognostic determinant in NSCLC. While immunochemotherapy offers incremental benefit, the persistent survival gap calls for innovative systemic and local interventions [45]. Future research should aim to transform MPE from a marker of poor prognosis into a manageable component of precision oncology. The evidence supports that while MPE severely impacts quality of life, available interventions can provide meaningful symptomatic relief, particularly for dyspnea, though more high-quality comparative studies are needed to optimize treatment selection [37,38].

## 7. Evaluating the Scale of Disease Burden: Pathologists’ Perspectives

Regardless of the sampling technique, material obtained from MPE can be classified as either small pleural tissue biopsies or pleural fluid aspirates (Table 1). These specimen types follow distinct processing pathways in pathology. Biopsy samples, typically obtained via thoracoscopy or image-guided procedures, consist of limited solid tissue that undergoes formalin fixation, paraffin embedding, and subsequent histological evaluation with hematoxylin-eosin staining and immunohistochemistry. In contrast, pleural fluid aspirates contain dispersed cells within a liquid medium and are processed through cytocentrifugation or cell block preparation, enabling cytomorphological assessment and ancillary studies such as immunocytochemistry and molecular testing. The choice of specimen type influences diagnostic yield and suitability for advanced analyses, including biomarker profiling and next-generation sequencing.

Histology and cytology are two critical disciplines in pathology, focused on examining tissue and cell samples, respectively, to diagnose diseases. Although both methods involve the analysis of biological specimens under a microscope, their sample preparation workflows are distinct, reflecting their different objectives and the nature of the material being studied. Both histology and cytology deliver consistent morphological and immunophenotypic results, allowing a correct classification of disease and a first evaluation of the probable biological behavior of neoplasia. However, neoplastic tissue material is today used not only for pure morphological diagnoses, but also as a source of neoplastic nucleic acids to be analyzed, profiled, and sequenced.

Concordance between different specimen types is a key parameter evaluated across multiple studies in the literature, and it is intended to highlight the key differences in sample quality and the efficiency of nucleic acid extraction in various sample types, as well as the effectiveness of these materials in downstream molecular analyses such as next-generation sequencing (NGS), PCR, and other diagnostic assays [46], demonstrating that molecular profiling of NSCLC in pleural effusion is possible in a real-world setting.

NGS panels should ideally cover clinically actionable alterations classified as ESCAT Tier I according to current ESMO recommendations [47], including substitutions, insertions and deletions (indels), copy number variants, gene fusions, and splice alterations across the principal driver genes relevant to NSCLC [47]. Histological and cytological revision and material selection appear to be a pivotal moment to ensure analytical performance.

Pre-analytical variables such as sample type, processing method, and collection timing and temperature play an essential role in preserving nucleic acid integrity and ensuring accurate molecular profiling. In a retrospective cohort of 44 patients with metastatic NSCLC-associated MPE, Carter et al. highlighted the significant impact of pre-analytical variables on molecular yield and mutation detection [48]. Cytoblock samples, while commonly used in traditional cytology, often undergo nucleic acid degradation due to the extensive processing involved in tissue fixation and paraffin embedding [49,50], complicating both routine diagnostic and research studies [51].

The workflows of histology and cytology differ not only in the nature of the samples but also in the complexity and time required for processing: histology is a more intricate process due to the need to preserve, process, section, and stain larger tissue samples. This workflow is more time-consuming and may take several days to complete. Cytology, on the other hand, generally involves simpler procedures and is more rapid. Cytology samples are often analyzed in a shorter time frame, sometimes within hours, making it ideal for quicker diagnoses in some clinical situations [52].

Moreover, the differences in reagents and methods of preparation lead to a different type of damage to nucleic acids, representing the main pre-analytical variable influencing molecular results [53,54]. Once the sample is collected, both histology and cytology require fixation to preserve the cells or tissues in their current state and prevent degradation. Tissue samples submitted for histology are typically fixed using formalin (formaldehyde solution), which preserves tissue architecture and cellular detail. Formalin penetration may take several hours to days, depending on the tissue size and density. In some cases, other fixatives like glutaraldehyde or ethanol may be used, depending on the specific needs of the study. For cytology specimens, fixation is usually achieved by spraying or immersing the sample in a fixative immediately after collection. Common fixatives include ethanol, methanol, or cytology-specific sprays. The fixation process is generally faster compared to histology, reflecting the smaller size and fewer components involved in cytology samples.

Formalin fixation is the step which accounts for most nucleic acid degradation, causing several types of sequence artifacts [55]:

(1) Crosslinks include protein–protein, protein–DNA, and DNA–formaldehyde adducts and interstrand DNA crosslinks. The cross-link with nearby histones induces conformational changes and ultimately DNA denaturation.
(2) Fragmentation is the most common type of damage, and it is linked to storage time and the pH of the fixation medium
(3) Abasic sites are commonly created after oxidization of formaldehyde to formic acid in an oxygen-rich atmosphere, causing hydrolysis of the N-bond of purine bases because polymerase incorporates adenosine into uracil, leading to false variants.
(4) Deamination of purine bases is a major problem of false SNV.
(5) Methylene bridge formation, via methiolation, incorporation of a formaldehyde group in the form of Methylol to a base and the following electrophilic attack, involves mainly adenine, thus creating problems with the correct annealing to poli(A) tail during cDNA synthesis

The literature supports MPE supernatant analysis as a valuable source for molecular profiling. A pilot study presented at the 3rd International Electronic Conference on Biomedicines (ECB2025) [56], conducted at the molecular biology laboratory of Bolzano Hospital (South Tyrol, Italy), evaluated the feasibility of effusion supernatant analysis in a preliminary cohort of 10 patients. The study reported 100% concordance with matched tissue samples and higher variant allele frequencies (VAF) compared to histological or cytological material. As these findings derive from an abstract-level report, they should be interpreted as preliminary, but they are consistent with retrospective peer-reviewed studies in this field. Supernatant material shows favorable analytical performance when compared with cellblocks in all molecular analyses, including NGS, in several studies [6,48]. In a retrospective study of 20 NSCLC cases, Tafoya et al. reported successful implementation of a 50-gene NGS panel using post-centrifuge supernatant cytology fluid [6]. More recently, Thakur et al., in a cohort of 72 patients, confirmed pleural effusion supernatant as a reliable source of cfDNA for molecular testing in lung cancer [8]. Similarly, Buttitta et al., analyzing 43 pleural fluid and bronchoalveolar lavage samples, demonstrated reliable EGFR mutation assessment using next-generation sequencing [57].

## 8. A Major Pitfall

Molecular profiling of MPEs has emerged as a valuable tool for guiding targeted therapy in advanced NSCLC. Techniques such as next-generation sequencing (NGS) applied to pleural fluid—whether from cell blocks, sediment DNA, or supernatant-derived cell-free DNA—offer high sensitivity and the potential to capture tumor heterogeneity. However, alongside these advantages, the risk of false positive results remains a critical concern that can mislead clinical decision-making [58]. False positives in MPE molecular analysis often stem from non-tumor DNA contamination. Germline variants and clonal hematopoiesis mutations can be mistakenly interpreted as somatic alterations, particularly when sequencing is performed on cell-free DNA from supernatants without paired normal controls. Yang et al. [59] highlighted this issue, recommending simultaneous sequencing of white blood cells to exclude non-tumor origin mutations. This phenomenon mirrors challenges observed in plasma liquid biopsies, where hematopoietic clones can confound the interpretation of actionable mutations.

Another contributor is pre-analytical variability. Low tumor cellularity (<10%) increases the likelihood of detecting background genetic material rather than true tumor-derived mutations. While NGS demonstrates superior sensitivity compared to Sanger sequencing in hypocellular samples, its deep sequencing capability can inadvertently amplify rare non-tumor alleles, leading to spurious calls. Studies by Leichsenring et al. [60] and Buttitta et al. [57] confirm that samples with minimal malignant cells are prone to both false negatives and false positives, underscoring the importance of cytologic assessment before molecular testing. NGS platforms, despite their robustness, are not immune to sequencing artifacts. Errors during library preparation, PCR amplification, or bioinformatic variant calling can generate false positives, particularly for low-frequency variants. Copy number variations (CNVs) pose an additional challenge; cfDNA fragmentation in supernatant samples limits CNV detection accuracy, and misinterpretation of noise as amplification events has been reported. False positive results can have profound consequences. Misclassification of resistance mutations such as EGFR T790M or ALK G1202R may lead to inappropriate therapy escalation, exposing patients to unnecessary toxicity and cost. Similarly, reporting incidental findings of uncertain significance raises ethical and legal concerns, especially when these variants influence treatment decisions without robust validation. To minimize false positives, a multi-pronged approach is essential:

- Paired normal DNA sequencing (e.g., leukocytes) to filter germline and hematopoietic variants.
- Rigorous cytologic triage to ensure adequate tumor cellularity before testing.
- Validation of NGS pipelines with stringent thresholds for variant allele frequency and depth of coverage.
- Orthogonal confirmation of clinically actionable mutations using alternative methods such as digital PCR or repeat testing on tissue when feasible.

While molecular profiling of MPEs offers a minimally invasive avenue for precision oncology, vigilance against false positives is imperative. Standardization of pre-analytical and analytical protocols, coupled with confirmatory testing, will safeguard the clinical utility of this promising diagnostic modality.

## 9. A Practical Tumor Board Workflow for Molecular Profiling in NSCLC with Malignant Pleural Effusion

The diagnostic and therapeutic pathway for molecular profiling in NSCLC with MPE must be adapted to the economic, organizational, and workforce characteristics of each clinical setting, while also reflecting the individual patient’s clinical needs. Variability in technological resources, personnel expertise, and institutional workflow requires a flexible and context-specific approach. Ensuring timely and clinically meaningful molecular testing therefore depends on aligning precision oncology standards with the operational realities of each center.

Multiple strategies can be adopted for selecting and integrating biological material for molecular profiling in the presence of MPE, including sequential, complementary, or liquid-first approaches. The choice among these pathways should be guided by clinical conditions, sample adequacy and quality, and therapeutic priorities. These approaches are consistent with recommendations from major international scientific societies, including The International Association for the Study of Lung Cancer (IASLC) [61] and The International Society of Liquid Biopsy (ISLB) [62], which emphasize the importance of dynamically incorporating tissue, cytology, and liquid biopsy samples to maximize diagnostic yield and support evidence-based decision-making. At present, there remains a lack of harmonization across key components of the testing pathway—including assay selection, procedural workflows, analytical techniques, and pre-analytical variables (e.g., collection methods, fixation, processing, and storage)—which can introduce variability in performance metrics and limit reproducibility across institutions. Nonetheless, international societies are actively working toward standardization, promoting consensus frameworks, minimum quality requirements, and best-practice recommendations aimed at reducing inter-laboratory variability and fostering robust, reproducible results that are transferable across clinical settings [63].

**Figure 3:**
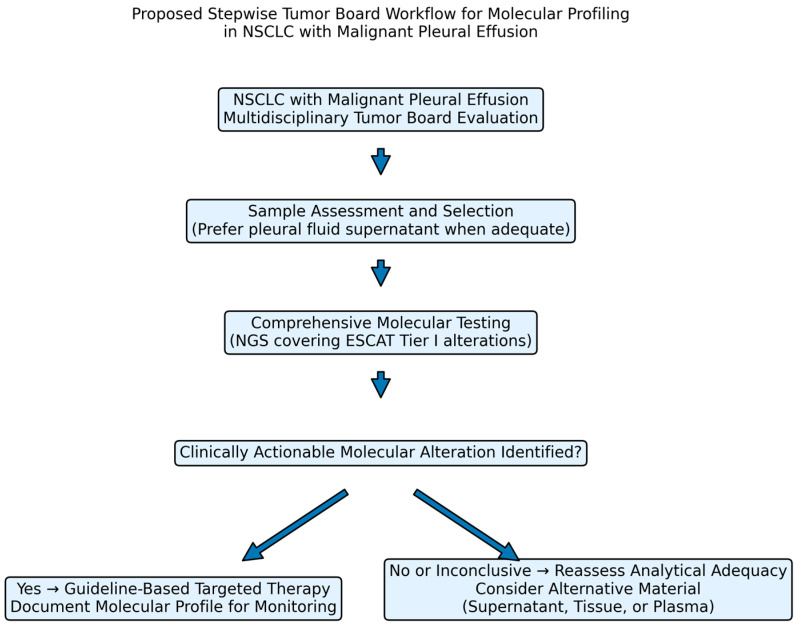
Proposed stepwise tumor board workflow for molecular profiling in NSCLC patients presenting with malignant pleural effusion. The algorithm illustrates a structured multidisciplinary approach integrating sample assessment, molecular testing, and predefined decision nodes. Following confirmation of NSCLC with malignant pleural effusion, available biological material is evaluated, with preference for analytically adequate pleural fluid fractions when feasible. Comprehensive next-generation sequencing is performed according to current guideline recommendations. Identification of a clinically actionable alteration leads to guideline-based targeted therapy, whereas negative or inconclusive results prompt reassessment of analytical adequacy and consideration of alternative material before establishing definitive molecular negativity.

To translate multidisciplinary discussion into a reproducible and clinically applicable strategy, we propose a structured yet pragmatic tumor board workflow for molecular profiling in patients with NSCLC presenting with MPE. This framework integrates clinical assessment, sample triage, minimum analytical requirements, test selection, and predefined contingency strategies in case of negative or insufficient results, thereby transforming conceptual collaboration into an operational pathway [47,60,61] (Figure 3).

At tumor board presentation, essential clinical parameters should be systematically documented, including confirmation or strong suspicion of metastatic NSCLC, the presence of MPE at diagnosis or progression, performance status, urgency of systemic treatment initiation, and availability of biological material such as pleural fluid, cytology preparations, small biopsies, or plasma. Early clarification of whether pleural fluid represents the primary or only available specimen is crucial to prevent delays in molecular testing and therapeutic decision-making [44,61].

Sample selection should be guided by pathological assessment and pre-analytical quality considerations. When available and technically feasible, pleural fluid supernatant should be strongly considered for molecular analysis, given its reduced formalin-induced nucleic acid damage and favorable variant allele frequencies reported in both pilot studies and larger series [6,55,56]. Fresh cytological preparations may provide adequate material for testing, whereas cytoblocks and small formalin-fixed biopsies remain valid but may be more susceptible to fixation-related artifacts affecting nucleic acid integrity [48,52,54]. Plasma liquid biopsy may serve as a complementary or fallback option when pleural material is unavailable or insufficient, consistent with IASLC recommendations for advanced NSCLC [61]. The choice among these materials should reflect not only availability but also anticipated analytical performance and clinical urgency.

Before molecular results are interpreted as definitive, minimum analytical criteria should be verified to ensure technical adequacy and clinical reliability. For cell-based specimens, cytological confirmation of malignant cells is essential, with an estimated tumor cellularity preferably of at least 10% to reduce the risk of false negative results, as supported by cytology-specific genomic handling guidelines and pleural fluid molecular studies [49,56,59]. DNA input should comply with platform-specific requirements, commonly in the range of 10–20 ng for targeted next-generation sequencing panels, and DNA quality must be compatible with library preparation [47]. Sequencing depth should be sufficient to enable reliable detection of low-frequency variants, typically with mean coverage of at least 500×, and variant allele frequency thresholds should align with validated laboratory standards, often around 1% depending on assay sensitivity and bioinformatic validation [47,61]. For pleural fluid supernatant-based testing, cytological confirmation of malignant effusion remains important to ensure clinical relevance, while adequate cfDNA yield and validated assay sensitivity for low variant allele frequencies are required [61,63]. Bioinformatic pipelines should incorporate safeguards to minimize sequencing artifacts and, when appropriate, distinguish tumor-derived variants from potential clonal hematopoiesis-related alterations [57,58,61]. If these analytical criteria are not fulfilled, results should be interpreted with caution, and alternative material should be considered before concluding true molecular negativity [63].

Test selection should be adapted to the clinical scenario, therapeutic urgency, and material adequacy. In newly diagnosed advanced NSCLC presenting with malignant pleural effusion, comprehensive next-generation sequencing panels are recommended, covering single-nucleotide variants, insertions and deletions, copy number variations, gene fusions, and splice alterations, and including all clinically actionable targets according to current ESCAT Tier I recommendations [47]. In cases of disease progression under targeted therapy, expanded or resistance-focused panels may be more appropriate, and tumor-informed liquid biopsy strategies can be considered when prior molecular data are available [42,61]. When DNA quantity is limited, a prioritized or sequential testing approach may be adopted, initially focusing on high-impact driver alterations with immediate therapeutic implications, followed by reflex expansion to broader profiling if material permits [47,61]. In situations requiring rapid therapeutic decision-making, targeted PCR or digital PCR assays for common actionable drivers may be performed as an expedited strategy, with comprehensive sequencing undertaken in parallel or immediately thereafter [61]. Importantly, a negative result derived from analytically suboptimal material should not be regarded as equivalent to confirmed molecular absence of actionable alterations [63].

Interpretation of molecular results should follow predefined decision nodes within the tumor board. Identification of a clinically actionable alteration should lead to guideline-based targeted therapy and documentation of the molecular profile as a reference for future resistance monitoring [47,61]. In contrast, negative or inconclusive results should prompt reassessment of analytical adequacy, including tumor representation and sequencing performance. If material quality or quantity was suboptimal, alternative biological fractions, plasma liquid biopsy, or, when clinically feasible, repeat biopsy should be considered before establishing a definitive molecularly negative status [61,63]. Through this structured yet flexible approach, malignant pleural effusion can be transformed from a traditionally palliative marker into an integrated component of precision oncology decision-making.

## 10. Conclusions

MPEs represent a clinically challenging condition, often associated with advanced disease and poor prognosis. Traditionally considered a palliative marker, MPEs pose significant therapeutic challenges as well as diagnostic difficulties, the latter due to limited tissue availability and heterogeneous cellular composition. Beyond their molecular implications, MPEs require coordinated clinical management, including radiological evaluation, symptom-directed interventions, and procedural strategies aimed at improving quality of life. However, recent advances in cytopathology, histopathology, and liquid-based techniques have demonstrated that material obtained from pleural effusions, whether in the form of cytological preparations, small biopsies, or cell-rich fluid, can provide critical molecular and immunohistochemical information. These findings underscore the potential of MPE-derived specimens to guide biomarker-driven strategies and enable precision oncology approaches, transforming a historically unfavorable clinical scenario into an opportunity for targeted therapy selection. The role of the cytologist/cytopathologist is central to the successful implementation of liquid biopsy in clinical practice, playing a pivotal role in selecting the appropriate biological material for molecular testing and optimizing pre-analytical handling to maximize diagnostic yield.

Incorporating liquid biopsy technology into routine clinical workflows requires careful consideration of several factors, including laboratory infrastructure, expertise in molecular biology, and the need for standardized protocols to ensure consistent and reproducible results. At the same time, optimal patient outcomes depend on integration of molecular findings with imaging assessment, procedural decision-making (e.g., pleurodesis or indwelling pleural catheter placement), and systemic treatment planning within the tumor board setting. The successful adoption of pleural fluid-based molecular testing depends on validated assays, adequate training, and familiarity with assay sensitivity, specificity, and pre-analytical variables. Consequently, although the growing body of evidence supports the feasibility and clinical relevance of this approach, implementation remains heterogeneous across institutions, particularly where technical resources or specialized personnel are limited.

Importantly, the strength of the current evidence base should also be interpreted with caution. Much of the available literature on molecular profiling of malignant pleural effusions derives from retrospective analyses, single-center experiences, and heterogeneous patient cohorts, often with variable pre-analytical workflows and sequencing platforms. Sample sizes in concordance and feasibility studies are frequently limited, and prospective multicenter validation remains scarce. Differences in assay design, analytical thresholds, and reporting standards may further restrict cross-study comparability. While pleural fluid-based molecular testing appears promising and clinically informative, these factors underscore the need for continued standardization and larger, prospective investigations to avoid overgeneralization and to support broader implementation.

Ultimately, the management of malignant pleural effusion in advanced NSCLC reflects a multidisciplinary precision oncology model, in which radiologists, pulmonologists, surgeons, pathologists, and oncologists collaborate to integrate diagnostic, procedural, and molecular information. Collaborative projects among different specialties help consolidate trust in the tumor board decision-making process, improving patients’ overall care delivery.

## Figures and Tables

**Table 1:** Comparative Analysis of Cytology and Histology in Pleural Effusion Diagnostics.

Parameter	Cytology		Histology
**Slide examples**	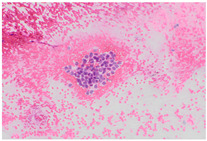 Smear cytology	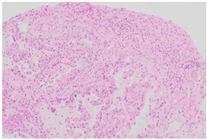 Cytoblock	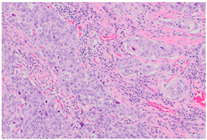 Histology
**Specimen Type**	Pleural fluid aspirates (cells dispersed in liquid)		Small pleural tissue biopsies
**Workflow** **Complexity**	Simple, rapid (often within hours)		Complex, multi-step (several days)
**Processing Steps**	Cytocentrifugation or cell block; ethanol/methanol fixation		Formalin fixation → paraffin embedding → sectioning → staining
**Turnaround Time**	Short (ideal for quick diagnosis)		Longer due to fixation and embedding
**Morphological Detail**	Excellent cellular detail; limited architectural context		Comprehensive tissue architecture and subtyping
**Molecular** **Profiling**	High feasibility; best results from supernatant cfDNA (minimal degradation)		Feasible but affected by formalin-induced DNA damage (crosslinks, fragmentation)
**Pre-analytical** **Issues**	Cytoblocks may degrade nucleic acids; supernatant preferred		Formalin fixation causes major DNA/RNA damage and sequence artifacts
**Advantages**	• Faster results • Minimally invasive • Better cfDNA integrity		• Full tissue architecture • Reliable immunohistochemistry
**Limitations**	• Limited architectural context • Cytoblock degradation risk		• Longer processing time • Higher risk of nucleic acid damage
**Ideal Use Case**	Rapid diagnosis and molecular testing from effusion supernatant		Confirmatory diagnosis and detailed histological subtyping

The histological images shown represent fully anonymized archival diagnostic material from routine pathology practice and are used solely for illustrative purposes.

## Data Availability

No new data was created.

## Abbreviations

The following abbreviations are used in this manuscript:

MPE	Malignant pleural effusion
NSCLC	Non-small cell lung cancer
TB	Tumor board
IPC	Indwelling pleural catheters
LOS	Length of stay
VATS	Video-assisted Thoracoscopic Surgery
MPM	Malignant Pleural Mesothelioma
ICI	Immune checkpoint inhibitor
MRD	Minimal residual disease
NGS	Next-generation sequencing
CNV	Copy number variation
IASLC	International Association for the Study of Lung Cancer
